# Representation of the His Bundle Cloud on a Three-dimensional Electroanatomical Map and Its Implications for His Bundle Pacing

**DOI:** 10.19102/icrm.2021.120507

**Published:** 2021-05-15

**Authors:** Helbert Acosta, Nathan J. Acosta, Gustavo Lopera

**Affiliations:** ^1^UnityPoint Health Heart Center, Rock Island, IL, USA; ^2^Orlando Veterans Affairs Medical Center, Orlando, FL, USA; ^3^University of Central Florida College of Medicine, Orlando, FL, USA

**Keywords:** His bundle pacing, three-dimensional electroanatomical mapping

## Abstract

His bundle pacing has been proposed as a more physiologic approach to chronic ventricular stimulation, yet the achievement of permanent His bundle pacing can be challenging.

His bundle (HB) pacing has been proposed as a more physiologic approach to chronic ventricular stimulation.^1^ However, the achievement of permanent HB pacing can be challenging. As the HB cannot be visualized, the recording of the HB potential (HBP) with a multipolar catheter has been used to guide the insertion of the lead at the HB for permanent pacing.^2^ One of the limitations of this approach is that recording the HBP does not guarantee HB capture. This is due to the fact that not all the sites at which an HBP is recorded at the atrioventricular junction correlate with the location of the HB body. This notion has led to the concept of the HB cloud.^2^

**Figure 1** depicts a three-dimensional electroanatomical map (Medical Precision Mapping System; Abbott, Chicago, IL, USA) of the right atrium (RA), the base of the right ventricle (RV), and the superior (SVC) and inferior vena cavae (IVC), obtained using a decapolar catheter for reference and a 4-mm-tip ablation catheter for mapping and pacing. Extensive and detailed bipolar mapping of the atrioventricular junction area was performed with the mapping catheter to record and pace the HB. Each site at which the HBP was recorded was color-tagged. Subsequently, bipolar pacing was attempted at each of the color-tagged sites (using double the amplitude of the local capture threshold at a 1-ms pulse width). The sites where selective and nonselective HB capture were achieved were marked with red and orange tags, respectively. The yellow tags represent sites where only the right ventricular tissue was captured despite the recording of an HBP.

This image report confirms the concept that the ability to record the HBP does not guarantee HB capture. Moreover, the amplitude of the HBP does not predict the ability to capture the HB, as depicted in **Figure 2**. The understanding of these concepts is of paramount importance when attempting HB pacing.

## Figures and Tables

**Figure 1:**
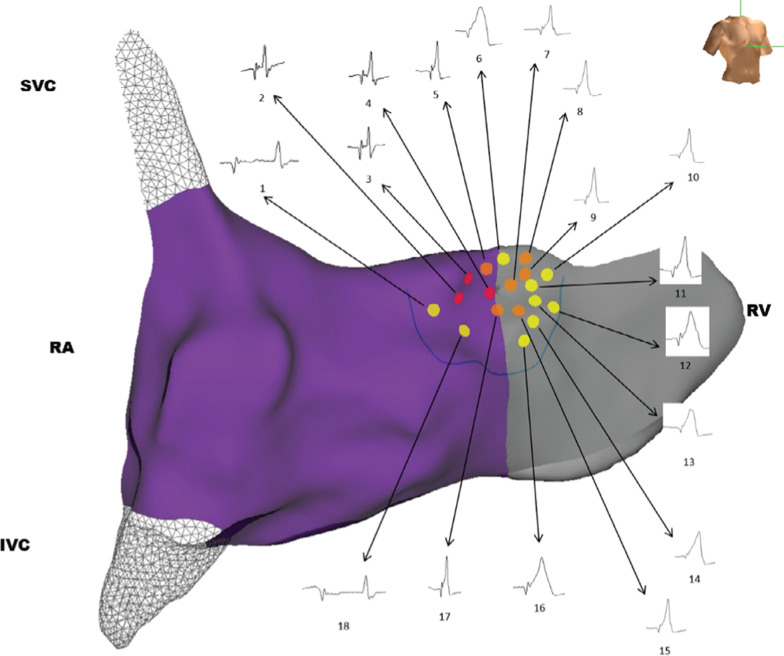
Three-dimensional electroanatomical map of the HB cloud. The sites where selective and nonselective HB capture was achieved are marked with red and orange tags, respectively. The yellow tags represent sites where only right ventricular tissue was captured despite recording of an HBP. IVC: inferior vena cava; RA: right atrium; RV, right ventricle; SVC: superior vena cava.

**Figure 2:**
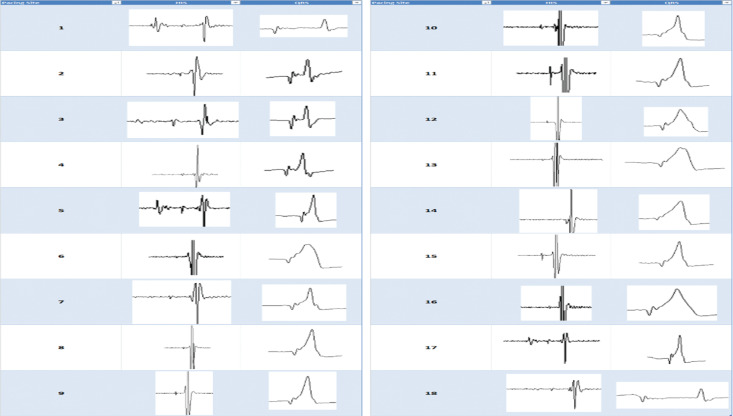
As demonstrated, the presence of a HB potential does not guarantee HB capture. Moreover, the amplitude of the HB potential does not predict the ability to capture the HB.
